# Gastrointestinal mixed adenoneuroendocrine carcinoma: a population level analysis of epidemiological trends

**DOI:** 10.1186/s12967-020-02293-0

**Published:** 2020-03-14

**Authors:** Jiakun Wang, Aoxiao He, Qian Feng, Ping Hou, Junjun Wu, Zhihao Huang, Zhouqing Xiao, Chi Sun, Wenjun Liao, Linquan Wu

**Affiliations:** 1 Department of General Surgery, The Second Affiliated Hospital of Nanchang University, No. 1, Minde Road, Nanchang, 330006 China; 2 Department of Emergency, The Second Affiliated Hospital of Nanchang University, No. 1, Minde Road, Nanchang, 330006 China; 3 Department of Nursing, The Second Affiliated Hospital of Nanchang University, No. 1, Minde Road, Nanchang, 330006 China

**Keywords:** Gastrointestinal mixed adenoneuroendocrine carcinoma (MANEC), Annual percent change (APC), Trend, Epidemiology, Prognosis

## Abstract

**Background:**

The rise in incidence and mortality of gastrointestinal mixed adenoneuroendocrine carcinoma (MANEC) has not been well focused. The aim of our study was to examine epidemiological trends in incidence and incidence-based (IB) mortality of gastrointestinal MANEC at a population level.

**Methods:**

The incidence and IB mortality of gastrointestinal MANEC as well as data on affected patients from 2000 to 2016 were obtained from the Surveillance, Epidemiology, and End Results database. Trends in incidence and IB mortality were assessed using Joinpoint regression. The Kaplan–Meier method and log-rank test were used for survival analysis. Cox proportional hazards regression was used to identify independent predictors of mortality.

**Results:**

581 patients diagnosed with gastrointestinal MANEC were enrolled. Gastrointestinal MANEC incidence was 0.23 cases per 1,000,000 individuals in 2000 and 1.16 cases per 1,000,000 individuals in 2016, with an annual percent change (APC) of 8.0% (95% CI 5.7–10.3%, P < 0.05). IB mortality also showed a sustained increase (APC 12.9%, 95% CI 9.0–16.8%, P < 0.05). In Cox regression analysis, age at diagnosis, tumor grade and stage, lymph node metastasis, surgery, and tumor size were independently associated with mortality. Median survival was 75 months (95% CI 60–128 months). Median survival of appendiceal MANEC was significantly longer than that of cecal MANEC (115 vs. 31 months; P < 0.001).

**Conclusions:**

We found a sustained and rapid increase both in incidence and IB mortality of gastrointestinal MANEC, manifesting that there has been no significant improvement in patient outcomes, nor progress in prevention and treatment. Additional resources should be devoted to gastrointestinal MANEC research.

## Background

Mixed adenoneuroendocrine carcinoma (MANEC) is a rare subtype of neuroendocrine neoplasm consisting of both adenocarcinomatous and neuroendocrine cells. Each component must account for at least 30% of the lesion [1]. Cordier first described gastrointestinal tumors containing epithelial and neuroendocrine components in 1924, and in the years since, many different terms have been used to describe this hybrid tumor [2]. These include composite glandular-neuroendocrine mixed tumor, mucin-producing carcinoid, composite carcinoid-adenocarcinoma, collision tumor, adenocarcinoma ex-goblet cell carcinoid and other names [3–7]. In the early stage, inconsistent nomenclature had confused clinicians and pathologists, and made this disease difficult to summarize. This phenomenon led to a lack of awareness of this disease, which eventually related to little attention.

Currently, MANEC diagnosis mainly relies on histopathological examination. When hematoxylin and eosin staining demonstrate the presence of both neuroendocrine and adenocarcinomatous components, MANEC should be suspected. The next step is to confirm the diagnosis with appropriate immunohistochemical methods, using at least two of the three pathological neuroendocrine markers (synaptophysin, chromogranin, and CD56) [1, 8–10].

MANEC is more common in gastrointestinal tract [3, 6]. However, a uniform treatment strategy for gastrointestinal MANEC is lacking and thus the condition represents a threat to population health. The limited information available from case reports and small single-center retrospective studies suggests that the main therapeutic strategies are surgery or confused chemoradiotherapy [5, 11–14]. However, this might be fragmentarily because of our limited understanding of the demographic, clinical and prognostic characteristics of patients with gastrointestinal MANEC [10, 11, 15–17]. The burden of gastrointestinal MANEC disease has been largely ignored as its epidemiology is poorly understood, and it has been treated as a rare disease. So the fact was unoptimistic.

The demographic, clinical and prognostic characteristics of patients with gastrointestinal MANEC remain unclear, limiting the ability of clinicians to treat these individuals. Understanding the epidemiological trends and prognostic features of gastrointestinal MANEC can help us to formulate more standardized treatment strategies and assess the clinical benefits, for example, early intervention or others. Therefore, the purpose of this study was to explore trends in the incidence and incidence-based (IB) mortality of gastrointestinal MANEC using the Surveillance, Epidemiology, and End Results (SEER) database and evoke more attention for this kind of disease. We also examined the independent predictors of mortality.

## Materials and methods

### Date source

The SEER database from the National Cancer Institute (NCI) is an authoritative source of follow-up information for cancer patients [18]. We used the SEER-18 database, derived from cancer registries representing approximately 28% of the U.S. population, to collect data from 2000 to 2016 on the incidence and IB mortality of gastrointestinal MANEC patient, as well as comprehensive characteristics of patients with this condition.

### Study population

**Fig. 1:**
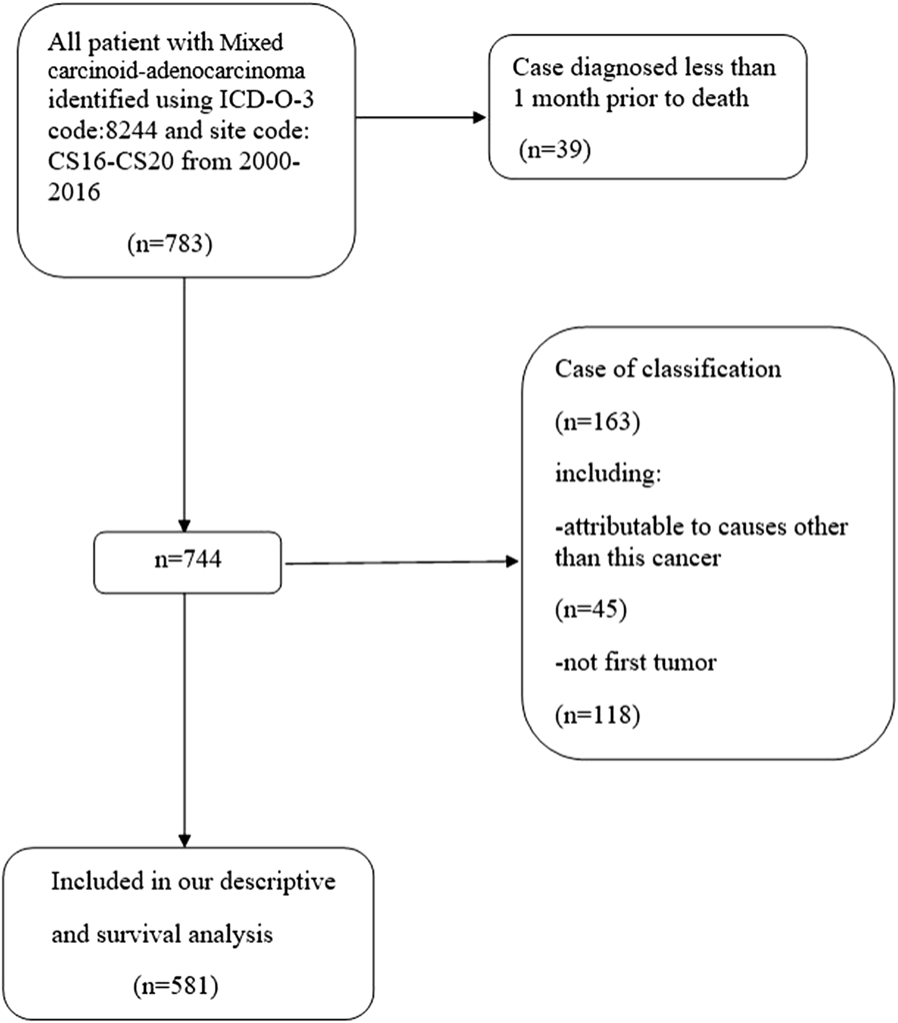
Flow diagram of patient selection out of the total 783 patients in the SEER database 2000–2016

International Classification of Disease for Oncology 3 (ICD-O-3) codes (8244) and site codes (C15–C20) were used to identify patients with gastrointestinal MANEC from 2000 to 2016 in the SEER database. Mixed carcinoid-adenocarcinoma is synonymous with MANEC in the SEER database. Because the minimum unit for survival was months and not day, data on patients who died within 1 month of diagnosis were excluded to avoid analyzing survival durations of zero. In addition, we excluded patients with another primary tumor in case these patients were misdiagnosed with gastrointestinal MANEC as a result of metastatic disease. Patients who died from causes other than gastrointestinal MANEC were also excluded (Fig. 1). We used the same method to obtain data on patients with carcinoid tumors and adenocarcinomas of the gastrointestinal tract for comparison. 293,043 patients with gastrointestinal adenocarcinomas and 26,223 patients with gastrointestinal carcinoid are included. We used the SEER stage classification, which provides consistent definitions over time, instead of the American Joint Committee on Cancer stage classification, which might change over the study period. The SEER stage classifications are as follows: localized stage (cancer confined to primary site), regional stage (cancer spread to regional lymph nodes), and distant stage (cancer spread to distant tissues and organs).

### Statistical analyses

SEER*Stat software (version 8.36) was used to obtain data on incidence, IB mortality, and patient characteristics. We analyzed IB mortality rather than overall mortality because death certificates do not include the histological information of the tumor [19]. In a given year, only a subset of total deaths was caused by gastrointestinal MANEC. Individuals defined as having died of gastrointestinal MANEC must have been diagnosed early rather than at autopsy. IB mortality was calculated by combining the incidence of tumors with death certificates. Incidence and IB mortality were age-adjusted to the standard population in the USA in 2000.

We used the Joinpoint Regression program (version 4.5.01) from the NCI to assess annual percent change (APC), which could demonstrate trends in incidence and IB mortality. APC is one approach to describe trends in incidence or mortality over time, and shows the slope, gradient or direction of each linear segment. In this way, tumor incidence or mortality is assumed to change at a constant percentage from the previous year using a model that is segmented in log-linear form. The program calculated 95% confidence intervals (CIs).

We performed a subgroup analysis based on the location of the lesion (appendix, cecum, and other). Demographic and clinical characteristics were summarized using descriptive statistics for each group. Chi square tests were used to compare categorical variables. Survival curves were estimated using the Kaplan–Meier method and compared using the log-rank test. Chi square tests were used to assess differences in the constituent ratio. In addition, we used Cox proportional hazard models to examine factors associated with mortality. All P values were two sided, and values of P < 0.05 were considered statistically significant. All statistical analyses were performed using STATA/SE (version 11.0).

## Results

### General characteristics of patients and tumors

A total of 783 individuals were diagnosed with MANEC of the gastrointestinal tract from 2000 to 2016. Of these individuals, 581 patients met the inclusion criteria and were enrolled in our study (Fig. 1). We divided the study population into three groups based on the location of the lesion (appendix, cecum, and other) (Table 1). The median age at diagnosis in the overall cohort was 59 years (interquartile range: 50–67 years). The proportions of men (n = 274, 52.8%) and women (n = 307, 47.2%) in the cohort were similar. The vast majority of gastrointestinal MANEC patients were Caucasian (n = 476, 81.2%). Most patients had regional gastrointestinal MANEC (n = 237, 40.4%), followed by distant disease (n = 185, 31.6%) and localized disease (n = 158, 27.0%). Most patients had poorly differentiated grade tumors (n = 209, 35.7%) and for a large proportion of patients the tumor grade was unknown (n = 191, 32.6%).

**Table 1:** Trends in baseline demographic and pathological characteristics of the study population (2000–2016)

Variable	Total	Appendix	Cecum	Other^a^
No. of patients (n)	581	354	56	171
Median age (years)	59	57	61.5	63
Gender, n (%)				
Women	274 (47.2)	176 (49.7)	25 (44.6)	73 (42.7)
Men	307 (52.8)	178 (50.3)	31 (55.4)	98 (57.3)
Race, n (%)				
White	472 (81.2)	307 (86.7)	44 (78.6)	121 (70.8)
Black	67 (11.5)	33 (9.3)	9 (16.1)	25 (14.6)
Other^b^	42 (7.3)	14 (4.0)	3 (5.3)	25 (14.6)
SEER historic stage, n (%)				
Localized	157 (27.0)	101 (28.5)	7 (12.5)	49 (28.7)
Regional	234 (40.3)	135 (38.1)	25 (44.6)	74 (43.3)
Distant	184 (31.7)	114 (32.2)	23 (41.1)	47 (27.5)
Unstaged	6 (1.0)	4 (1.1)	1 (1.8)	1 (0.5)
Grade, n (%)				
Well differentiated	52 (9.0)	32 (9.0)	1 (1.8)	19 (11.1)
Moderately differentiated	88 (15.1)	45 (12.7)	8 (14.3)	35 (20.5)
Poorly differentiated	205 (35.3)	125 (35.3)	32 (57.1)	48 (28.1)
Undifferentiated	46 (7.9)	15 (4.3)	6 (10.7)	2514.6)
Unknown	190 (32.7)	137 (38.7)	9 (16.1)	44 (25.7)

^a^Other group include: stomach:30 individuals, Duodenum:11 individuals, Jejunum:1 individual, Ileum:11 individuals, Small intestine:5 individuals, Ascending colon:24 individuals, Transverse colon:10 individuals, Descending colon:2 individuals, Sigmoid colon:20 individuals, Overlapping lesion of colon:5 individuals, Colon:4 individuals, Rectosigmoid junction:3 individuals, Rectum:45 individuals

^b^Other race include: American Indian/Alaskan Native, Asian/Pacific Islander

The median age at diagnosis in patients with appendiceal and cecal MANEC was 57 years (interquartile range: 49–64 years) and 61.5 years (interquartile range: 56–67 years), respectively. Caucasians formed the bulk of both appendiceal MANEC (n = 307, 86.7%) and cecal MANEC (n = 44, 78.6%) patients. It is interesting to note that the proportion of patients with localized appendiceal MANEC patients was higher than that with localized cecal MANEC (n = 101, 28.5% vs. n = 7, 12.5%; p < 0.001). However, the proportion of patients with poorly differentiated MANEC of the appendix was lower than that with poorly differentiated MANEC of the cecum (n = 125, 35.3% vs. n = 32, 57.1%; p = 0.002). The results are shown in Table 1.

Distant disease was more common in patients who did not undergo surgery than in patients who did (76.4% vs. 28.5%, P < 0.001) (Additional file 1: Fig. S1a). Conversely, localized disease was more common in patients who underwent surgery than in patients who did not (28.2% vs. 10.5%, P = 0.018). In addition, we also studied the relationships between tumor size and lymph node metastasis and SEER stage. The proportion of lymph node positivity in patients with tumors > 2 cm was higher than that in patients with tumors ≦ 2 cm (65.8% vs. 35.9%, P < 0.001) (Additional file 1: Fig. S1b). Distant gastrointestinal MANEC was more common in patients with tumors > 2 cm (35.1% vs. 12.0%, P < 0.001) and localized gastrointestinal MANEC was more common in patients with tumors ≦ 2 cm (48.9% vs. 14.0%, P < 0.001) (Additional file 1: Fig. S1c).

### Overall incidence and mortality trends

**Fig. 2:**
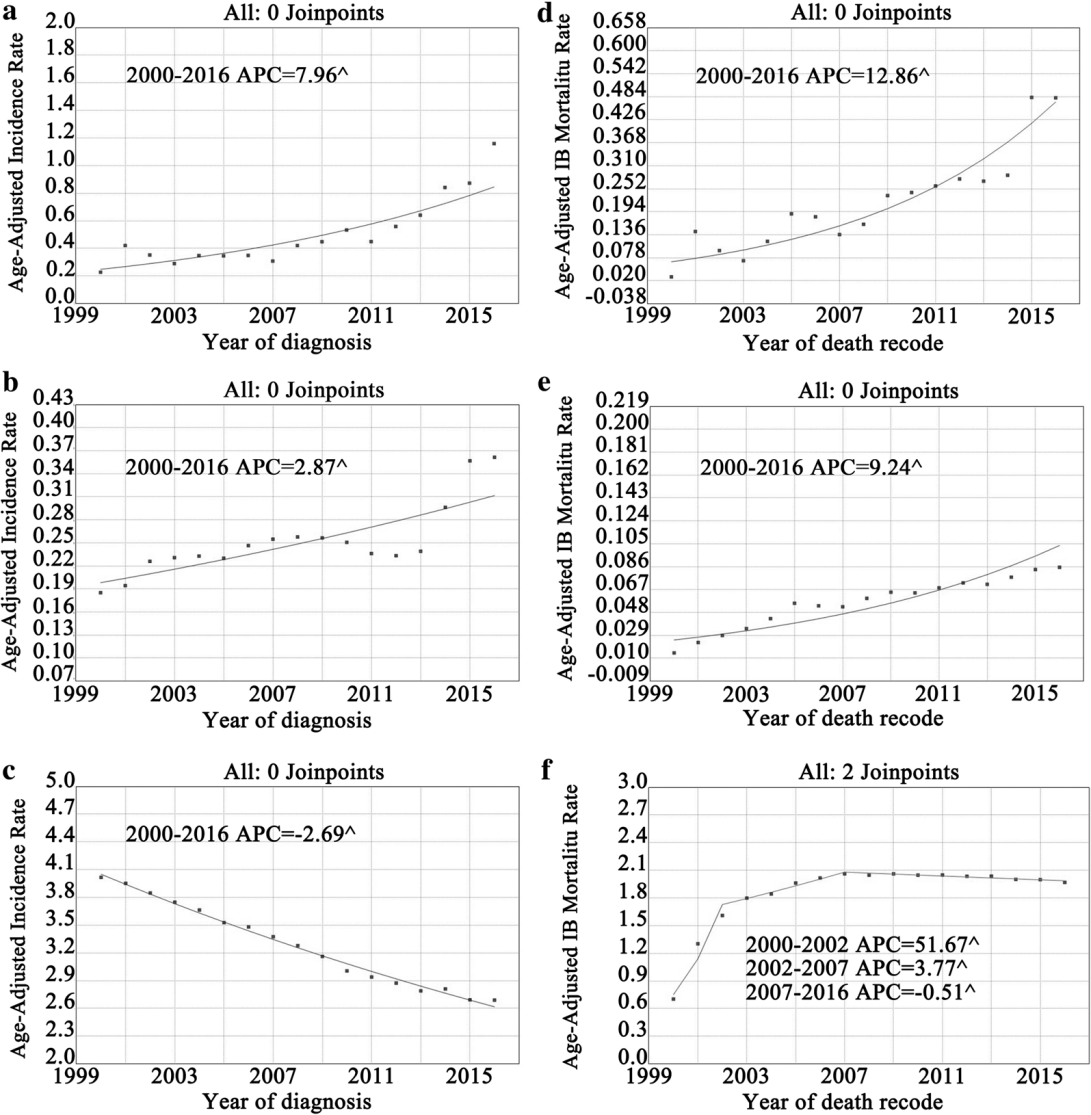
Incidence and IB mortality trends in gastrointestinal MANEC, carcinoid and adenocarcinoma overall 2000–2016. **a** Incidence trends in gastrointestinal MANEC. **b** Incidence trends in gastrointestinal carcinoid. **c** Incidence trends in gastrointestinal adenocarcinoma. **d** IB mortality trends in gastrointestinal MANEC. **e** IB mortality trends in gastrointestinal carcinoid. **f** IB mortality trends in gastrointestinal adenocarcinoma. ^ mean that P < 0.05

During the study period, the incidence of MANEC of the gastrointestinal tract showed continuous growth (Fig. 2a). The incidence of gastrointestinal MANEC was 0.23 cases per 1,000,000 individuals in 2000, and 1.16 cases per 1,000,000 individuals in 2016. The APC (i.e., the slope or extent of the increase in incidence) over this period was 8.0% (95% CI 5.7–10.3%, P < 0.05). Similarly, the incidence of gastrointestinal carcinoid tumors also showed a sustained increase (Fig. 2b). The incidence of the disease was 0.19 cases per 10,000 individuals in 2000, and 0.36 cases per 10,000 individuals in 2016. The APC was 2.9% (95% CI 1.8–4.0%, P < 0.05). Conversely, the incidence of gastrointestinal adenocarcinoma displayed a continuous decrease (Fig. 2c). The incidence of adenocarcinoma was 4.02 cases per 10,000 individuals in 2000, and 2.69 cases per 10,000 individuals in 2016. The APC was − 2.7% (95% CI − 2.8 to 2.5%, P < 0.05).

Gastrointestinal MANEC showed a sustained increase in IB mortality over the study period (Fig. 2d), with an APC of 12.9% (95% CI 9.0–16.8%, P < 0.05). The IB mortality of gastrointestinal carcinoid tumors also increased over the study period, with an APC of 9.2% (95% CI 6.8–11.7%, P < 0.05) (Fig. 2e). The IB mortality of adenocarcinomas of the gastrointestinal tract increased rapidly in the early study period (Fig. 2f), from 0.71 cases per 10,000 individuals in 2000 to 1.61 cases per 10,000 individuals in 2002. The APC was 51.7% (95% CI 44.8–55.6%, P < 0.05). However, the rise in adenocarcinoma IB mortality began to slow in 2002, with an APC of 3.8% (95% CI 2.9–4.6%, P < 0.05), and began to gradually decline in 2007, with an APC of − 0.5% (95% CI − 0.7 to − 0.3%, P < 0.05).

### Trend by sex

When the study population was stratified by gender, we found that the incidence of gastrointestinal MANEC rose in both men and women from 2000 to 2016 (Additional file 2: Fig. S2a). Incidence was slightly higher in males than in females. Moreover, the APC was 7.5% (95% CI 4.4–10.6%, P < 0.05) in men and 9.0% (95% CI 6.1–12.0%, P < 0.05) in women. The IB mortality of gastrointestinal MANEC followed a similar pattern in both men and women (Additional file 2: Fig. S2b). The APC in IB mortality was 13.3% (95% CI 9.0–17.8%, P < 0.05) in men and 13.5% (95% CI 8.0–19.4%, P < 0.05) in women over the study period.

### Trend by SEER stage

We next stratified the study population according SEER stage. Overall, the incidence of regional MANEC of the gastrointestinal tract was the highest and that of localized disease was the lowest among all three subgroups (Additional file 3: Fig. S3a). Changes in the incidence of regional and distant disease were similar, occurring at APCs of 9.77% (95% CI 6.1–13.6%, P < 0.05) and 9.82% (95% CI 6.3–13.4%, P < 0.05), respectively. The IB mortality of distant disease was increasing faster than that of regional disease (Additional file 3: Fig. S3b), with APCs of 13.4% (95% CI 9.4–17.5%, P < 0.05) and 11.1% (95% CI 4.6–8.0%, P < 0.05), respectively.

### Trend by lesion site

**Fig. 3:**
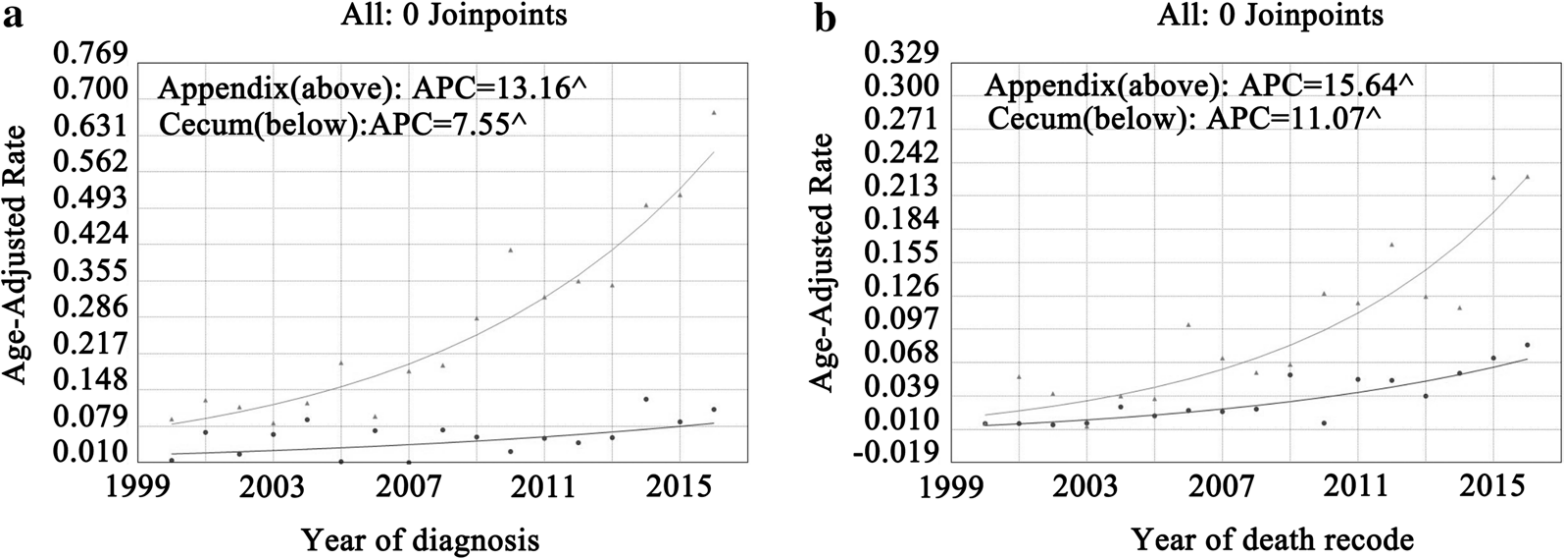
Incidence and IB mortality trends in appendiceal and cecal MANEC overall 2000–2016. **a** Incidence trends in appendiceal and cecal MANEC. **b** IB mortality trends in appendiceal and cecal MANEC

Overall, the incidence of appendiceal MANEC was higher than that of cecal MANEC, and both displayed a continuous increase (Fig. 3a). The APC of appendiceal MANEC was 13.2% (95% CI 10.3–16.1%, P < 0.05) and that of cecal MANEC was slightly lower. Similarly, IB mortality of both appendiceal and cecal MANEC showed sustained increases (Fig. 3b), with APCs of 15.6% (95% CI 10.5–21.0%, P < 0.05) and 11.1% (95% CI 7.4–14.9%, P < 0.05), respectively.

### Long-term survival outcomes

**Fig. 4:**
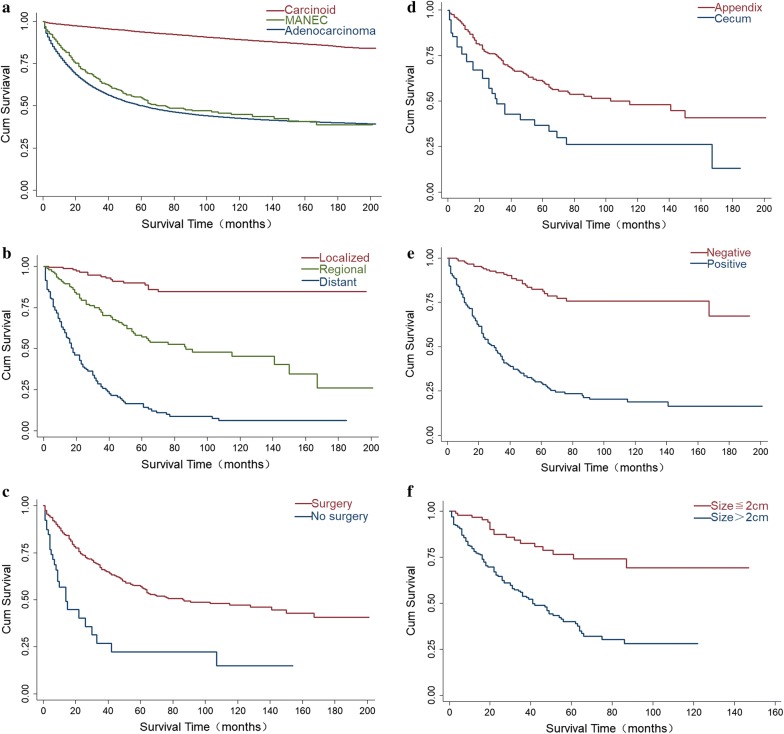
Long-term survival outcomes using Kaplan–Meier’s analysis: **a** long-term survival outcomes in gastrointestinal MANEC, carcinoid and adenocarcinoma. The survival was better in gastrointestinal carcinoid (p < 0.001) and worsen in gastrointestinal adenocarcinoma (p = 0.0167) compared with gastrointestinal MANEC. **b** Long-term survival outcomes in localized, regional and distant gastrointestinal MANEC. Graph shows increasing survival from localized to distant (p < 0.001). The P values reported for survival analysis refers to comparison among all stage. **c** Long-term survival outcomes in treatment of gastrointestinal MANEC (p < 0.001). **d** Long-term survival outcomes in appendiceal and cecal MANEC (p < 0.001). **e** Long-term survival outcomes in gastrointestinal MANEC results of lymph node examination (p < 0.001). **f** Long-Term Survival Outcomes in tumor size of gastrointestinal MANEC (p < 0.001)

The median survival of patients with gastrointestinal MANEC was 75 months (95% CI 60–128 months), and the 1-, 5-, and 10-year survival rates were 83.6%, 54.9%, and 44.9%, respectively. There was an obvious survival improvement in patients with gastrointestinal carcinoid tumors and a statistically significant decrease in survival of patients with gastrointestinal adenocarcinoma over the same period (Fig. 4a). In terms of SEER stage (Fig. 4b), patients with localized disease had the best prognosis and median survival, and patients with distant disease had shorter survival times than patients with regional disease (18 vs. 87 months, P < 0.001). The median survival of patients with well-differentiated tumors was significantly higher than that of patients with poorly-differentiated and undifferentiated tumors (Additional file 4: Fig. S4a). However, the median survival of patients with well-differentiated tumors was significantly higher than that of patients with poorly- differentiated and undifferentiated tumors. Moreover, patients who underwent surgery had better prognoses than those who received conservative treatment (median survival 15 vs. 86 months, P < 0.001). Moreover, patients who underwent surgery had better prognoses than those who received conservative treatment (median survival 15 vs. 86 months, P < 0.001) (Fig. 4c). Interestingly, we found that median survival of patients with lesions of the cecum was significantly lower than that of patients with lesions of the appendix (31 vs. 115 months, P < 0.001) (Fig. 4d). In addition, a statistically significant amelioration in survival was also observed in patients whose regional lymph nodes were negative (Fig. 4e) and whose tumors were > 2 cm (Fig. 4f). Conversely, there were no statistically significant differences in median survival between patients of different genders and races (Additional file 4: Fig. S4b, c).

Using Cox regression analysis, we found that age at diagnosis (> 60 years), SEER stage (regional and distant), tumor grade (poorly differentiated and undifferentiated), regional lymph node positivity, surgery, tumor size (> 2 cm), and cecal tumor site were all independently associated with gastrointestinal MANEC mortality (Table 2).

**Table 2:** Univariate Cox’s proportional hazards model assessing factors associated with mortality after diagnosis of mixed adenoneuroendocrine carcinoma in the gastrointestinal tract

Risk factor	Hazard ratios (HR)^a^	95% CI		P value
		Lower	Upper	
Age at diagnose (years)				
≦ 60	Referent			
> 60	1.52	1.17	1.98	0.002
Race				
Other	Referent			
White	1.87	0.96	3.66	0.07
Black	2.07	0.98	4.37	0.05
SEER stage				
Localized	Referent			
Regional	4.96	2.89	8.53	< 0.001
Distant	16.91	9.95	28.73	< 0.001
Treatment				
Surgery	Referent			
No surgery	3.01	1.97	4.62	< 0.001
Grade				
Well differentiated	Referent			
Moderately differentiated	1.93	0.93	3.99	0.08
Poorly differentiated	4.49	2.34	8.65	< 0.001
Undifferentiated	3.39	1.51	7.62	0.003
Regional lymph nodes				
Negative	Referent			
Positive	6.53	4.37	9.75	< 0.001
Tumor size				
≦ 2 cm	Referent			
> 2 cm	3.62	2.20	5.97	< 0.001
Tumor site				
Appendix	Referent			
Colon	1.81	1.22	2.69	0.003
Cecum	2.11	1.43	3.13	< 0.001

^a^HRs greater than 1.0 indicate a higher risk of death

Female gender, age at diagnosis (> 60 years), SEER stage (regional and distant), regional lymph node positivity, and tumor size (> 2 cm) were all independently associated with appendiceal MANEC mortality. Tumor grade (well- and moderately-differentiated) was associated with decreased cecal MANEC mortality. Regional lymph node positivity was independently associated with cecal MANEC mortality (Additional file 5: Table S1).

## Discussion

Because MANEC is a rare condition, and its definition and classification have only recently been solidified by the World Health Organization, the demographic characteristics and clinical prognoses of patients with gastrointestinal MANEC have not been adequately studied. Sporadic retrospective studies have addressed MANEC in the clinic, but these have generally been single-center studies with small sample sizes [10, 11]. The only available population-based analysis was confined to appendiceal MANEC: Brathwaite evaluated the clinical behaviors and prognoses of MANEC patients using a population-based database [20]. However, the incidence and mortality of gastrointestinal MANEC remain unclear, including changes over time. Therefore, describing trends in gastrointestinal MANEC incidence and mortality could help clinicians treat this disease more effectively.

The incidence of gastrointestinal MANEC showed a sustained increase in the US population over the study period. The rate of increase in incidence was steady at approximately 8.0% per year, faster than that of adenocarcinomas or carcinoid tumors of the gastrointestinal tract. The sustained increase in gastrointestinal MANEC incidence might reflect the fact that prevention measures have not improved much in recent years. Another explanation could be the development of new pathological techniques. We also analyzed adenocarcinomas and carcinoid tumors of the gastrointestinal tract for comparison. The incidence of gastrointestinal carcinoid tumors also increased during the study period. However, the APC of gastrointestinal carcinoid tumor incidence was lower than that of gastrointestinal MANEC (2.9% vs. 8.0%). Conversely, the incidence of gastrointestinal adenocarcinoma decreased over the study period. Compared with gastrointestinal adenocarcinoma or carcinoid tumors, interventions for gastrointestinal MANEC were not done well. Many more patients were affected by gastrointestinal adenocarcinoma or carcinoid tumors, and thus treatment of these conditions has attracted more attention. The increase rate of gastrointestinal MANEC incidence was much higher than expected, and a serious concern.

Gastrointestinal MANEC IB mortality also showed a sustained increase over the study period, with an APC of 12.9%. Thus, treatment and intervention programs for gastrointestinal MANEC are clearly unsatisfactory. In contrast, the IB mortality of gastrointestinal adenocarcinoma increased in the early years of the study period but showed a decreasing trend more recently. In addition, the APC of gastrointestinal carcinoid tumor IB mortality was much slower than that of gastrointestinal MANEC IB mortality, despite both IB mortalities showing increasing trends. This finding suggests that treatment and management of gastrointestinal adenocarcinomas and carcinoids have improved in recent years, especially for adenocarcinomas. Conversely, treatments for gastrointestinal MANEC have made little progress over the same period. Hence, more resources should be devoted to gastrointestinal MANEC and efforts should be made to develop improved prevention and treatment strategies.

Consistent with previous studies, gastrointestinal MANEC at the localized stage had a better prognosis with prolonged survival compared with distant disease [20–22]. Nevertheless, the incidence of distant gastrointestinal MANEC was higher than that of localized disease and had a higher APC, indicating that early diagnosis of gastrointestinal MANEC is unsatisfactory, and the incidence of distant disease is still predominant. In addition, survival was significantly longer in patients undergoing surgery over the study period. However, many patients are ineligible for surgery because the disease is too advanced at diagnosis. In our study, distant disease was more common in patients who did not undergo surgery than in patients who underwent surgery (76.4% vs. 28.5%, P < 0.001). Therefore, further efforts should concentrate on improving diagnosis and treatment in the early stages of disease, resulting in better survival benefits. Integrated treatment of advanced disease is also crucial and cannot be ignored.

In addition to demographic characteristics, we also examined survival of gastrointestinal MANEC. The prognoses of patients with gastrointestinal MANEC were significantly worse than those of patients with gastrointestinal carcinoid tumors. However, the median survival of patients with gastrointestinal MANEC was higher than that of patients with gastrointestinal adenocarcinoma. Carcinoid tumors are less aggressive with a 5-year survival rate of 88.7% according to some reports. Some studies even suggested that the 10-year survival rate for patients with positive lymph nodes was as high as 91% [23–25]. Adenocarcinomas are aggressive tumors commonly identified in the gastrointestinal tract and are prone to distant and lymph node metastasis [26, 27]. Owing to inadequate understanding, MANEC is usually treated like its less aggressive counterpart, carcinoid tumors. However, our findings indicate that gastrointestinal MANEC is a more aggressive tumor, with a prognosis intermediate between gastrointestinal adenocarcinoma and carcinoid tumors. Contrary to our findings, La Rosa et al. [12] found that there was no statistical difference in the median survival of patients with gastrointestinal MANEC and carcinoid tumors. Moreover, Wang et al. [28] found that MANEC had a worse survival than adenocarcinoma in gastrointestinal tract. These conflicting results may be due to small sample sizes: both studies enrolled only 12 MANEC patients. Our study was based on national population data, and a much larger sample size.

Our results showed that median survival of appendiceal MANEC was significantly longer than that of cecal MANEC. One potential explanation of this finding is that the distributions of tumor stage and grade at different lesion sites were different. The proportion of localized disease in appendiceal MANEC was significantly higher than that in cecal MANEC (P < 0.001). Furthermore, the proportion of poorly differentiated disease in appendiceal MANEC was significantly lower than that in cecal MANEC (P = 0.002). This difference in prognosis in patients with different lesion sites is crucial for management and treatment decisions.

In our analysis, distant gastrointestinal MANEC was more common in patients with tumors > 2 cm than in patients with tumors ≦ 2 cm (35.1% vs. 12.0%, P < 0.001). Conversely, localized gastrointestinal MANEC was more common in patients with tumors ≦ 2 cm than in patients with tumors > 2 cm (48.9% vs. 14.0%, P < 0.001). These findings suggest that tumor size may affect the biological behavior of gastrointestinal MANEC. Similarly, the proportion of lymph node positivity in patients with tumors > 2 cm was higher than that in patients with tumors ≦ 2 cm (65.8% vs. 35.9%, P < 0.001), which also indirectly suggests that tumors larger than 2 cm might have more invasive biological behavior.

We found that age > 60 years, regional and distant stage, poorly differentiated and undifferentiated tumor grade, positive lymph nodes, and tumor size > 2 cm were all independently associated with increased risk of death. These predictors of poor prognosis might inform clinical treatment decisions and support risk assessment.

Ours is the first study to use a population-based database to examine gastrointestinal MANEC demographic and prognostic characteristics, and it possessed the largest sample size to date. However, there were several inevitable limitations in our study. For instance, the SEER database does not provide information regarding the immunohistochemistry of the disease or the complications in each patient.

## Conclusions

The incidence and IB mortality of gastrointestinal MANEC showed a sustained increase in recent years, indicating that there has been no significant improvement in prevention and treatment. Therefore, we needed to realize the harmfulness of gastrointestinal MANEC and paid more attention. It could help us to understand this disease fully and formulate more standardized treatment strategies. In addition, we also found that some risk facts were independently associated with mortality. So, we believed that sufficient knowledge and early surgical intervention might be useful. Of course, more researches focus on gastrointestinal MANEC are needed.

## Supplementary information

**Additional file 1: Figure S1.** The constituent ratio of patients in gastrointestinal MANEC. (a) Stage of Gastrointestinal MANEC at diagnosis by treatment. (b) Lymph node examination result of Gastrointestinal MANEC by tumor size. (c) Stage of Gastrointestinal MANEC at diagnosis by tumor size. *mean that p < 0.05, **mean that p < 0.001.

**Additional file 2: Figure S2.** Incidence and IB mortality trends in gastrointestinal MANEC incidence trends from 2000–2016 for men and women. (a) Incidence trends in gastrointestinal MANEC for men and women, respectively. (b) IB mortality trends in Gastrointestinal MANEC for men and women, respectively. ^ mean that P < 0.05.

**Additional file 3: Figure S3.** Incidence and IB mortality trends in Gastrointestinal MANEC incidence trends from 2000–2016 for all stage. (a) Incidence trends in Gastrointestinal MANEC for all stage, respectively. (b) IB mortality trends in Gastrointestinal MANEC for all stage, respectively. ^ mean that P < 0.05. (The present of linear trends in IB mortality of localized disease was unavailable because the IB mortality of localized gastrointestinal MANEC was zero in some of years. So, we present it with scatter).

**Additional file 4: Figure S4.** Long-Term Survival Outcomes using Kaplan–Meier’s analysis: (a) Long-Term Survival Outcomes in grade of gastrointestinal MANEC. Graph shows no difference in median survival between well and moderately differentiated disease (p = 0.069), difference in median survival between well and poorly differentiated disease (p < 0.001), difference in median survival between well and undifferentiated disease (p = 0.0017). (b) Long-Term Survival Outcomes in race of gastrointestinal MANEC. Graph shows no difference in median survival between white, black and other. (p = 0.136) (c) gender of gastrointestinal MANEC (no statistically significant difference). Graph shows no difference in median survival between male and female (p = 0.173).

**Additional file 5: Table S1.** Univariate cox’s proportional hazards model assessing factors associated with mortality after diagnosis of mixed adenoneuroendocrine carcinoma in appendix and cecum.

## Data Availability

The data sets used or analyzed in this study are available from the corresponding author on reasonable request. We have been authorized by the SEER database.

## Abbreviations

MANEC: Mixed adenoneuroendocrine carcinoma
IB mortality: Incidence-based mortality
SEER: Surveillance, Epidemiology, and End Results
APC: Annual percent change
