# Supplementary figures and images for: Gastrointestinal mixed adenoneuroendocrine carcinoma: a population level analysis of epidemiological trends
Source: J Transl Med. 2020 Mar 14;18:128. doi: 10.1186/s12967-020-02293-0 (PMC7071749; doi:10.1186/s12967-020-02293-0)

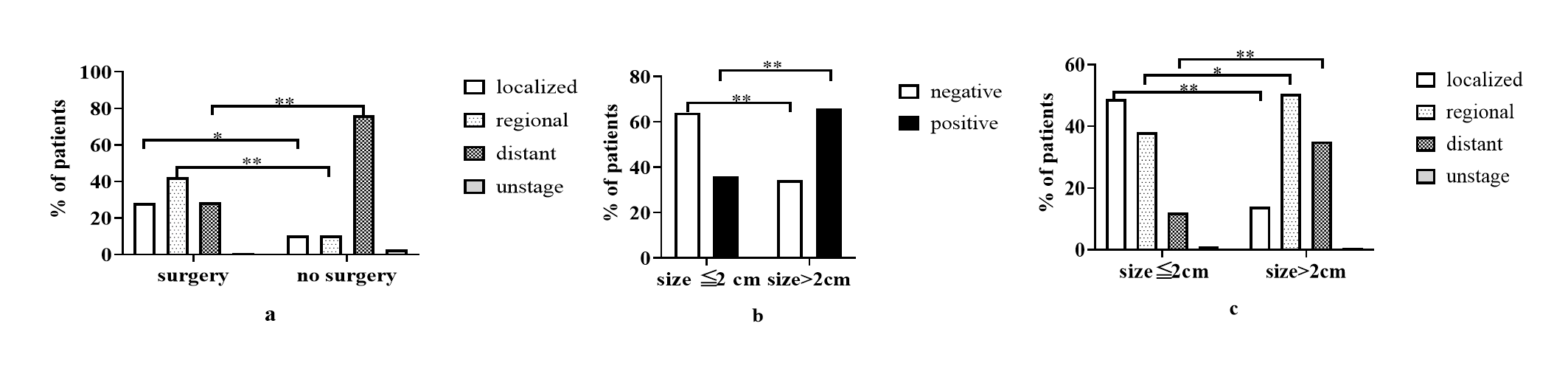

Supplement: Supplementary file 1 — Additional file 1: Figure S1. The constituent ratio of patients in gastrointestinal MANEC. (a) Stage of Gastrointestinal MANEC at diagnosis by treatment. (b) Lymph node examination result of Gastrointestinal MANEC by tumor size. (c) Stage of Gastrointestinal MANEC at diagnosis by tumor size. *mean that p < 0.05, **mean that p < 0.001. [file 12967_2020_2293_MOESM1_ESM.tif]

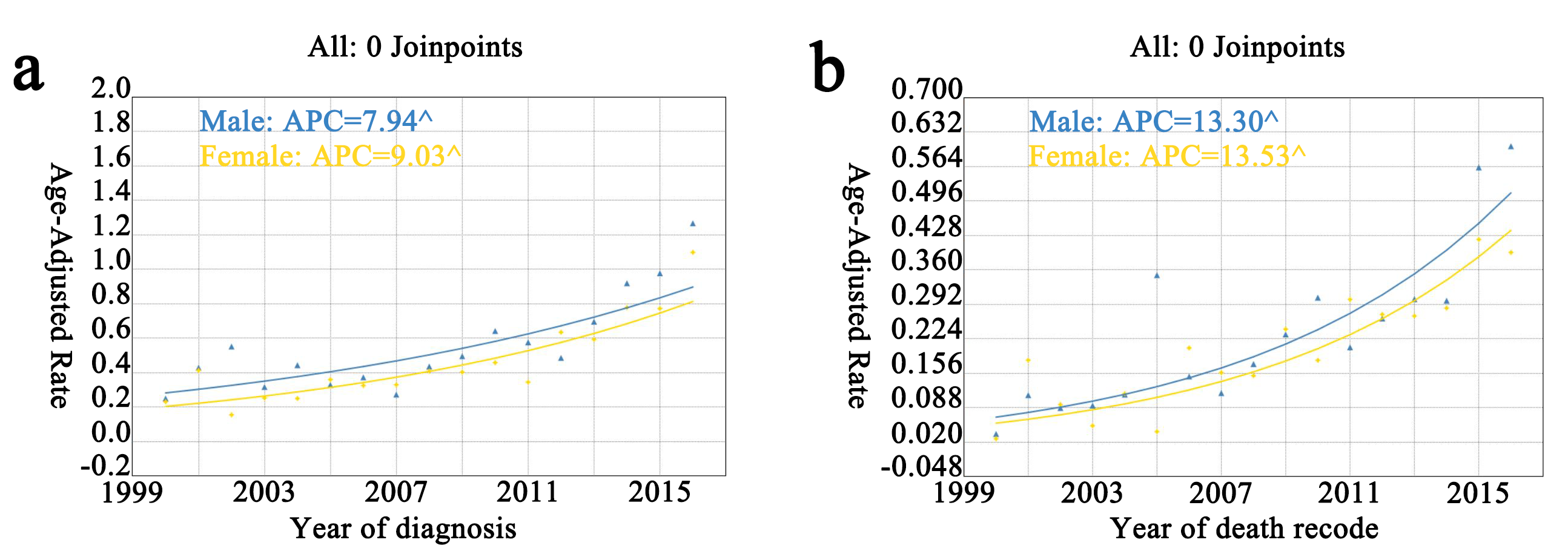

Supplement: Supplementary file 2 — Additional file 2: Figure S2. Incidence and IB mortality trends in gastrointestinal MANEC incidence trends from 2000–2016 for men and women. (a) Incidence trends in gastrointestinal MANEC for men and women, respectively. (b) IB mortality trends in Gastrointestinal MANEC for men and women, respectively. ^ mean that P < 0.05. [file 12967_2020_2293_MOESM2_ESM.tif]

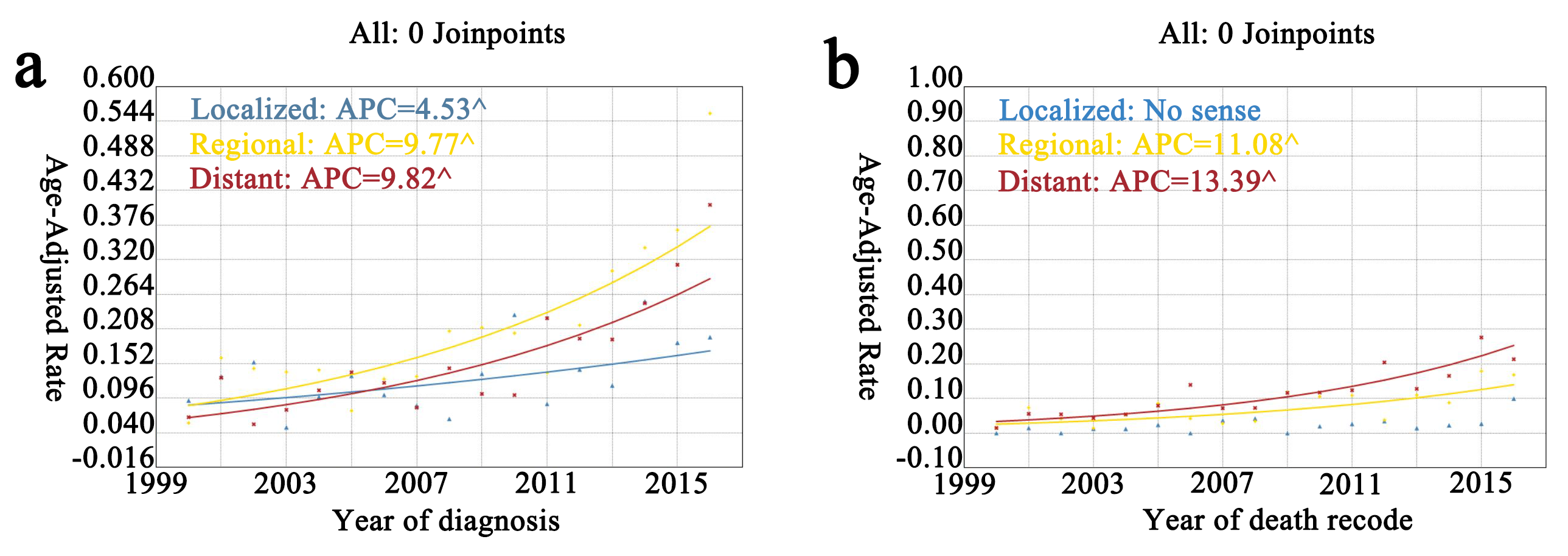

Supplement: Supplementary file 3 — Additional file 3: Figure S3. Incidence and IB mortality trends in Gastrointestinal MANEC incidence trends from 2000–2016 for all stage. (a) Incidence trends in Gastrointestinal MANEC for all stage, respectively. (b) IB mortality trends in Gastrointestinal MANEC for all stage, respectively. ^ mean that P < 0.05. (The present of linear trends in IB mortality of localized disease was unavailable because the IB mortality of localized gastrointestinal MANEC was zero in some of years. So, we present it with scatter). [file 12967_2020_2293_MOESM3_ESM.tif]

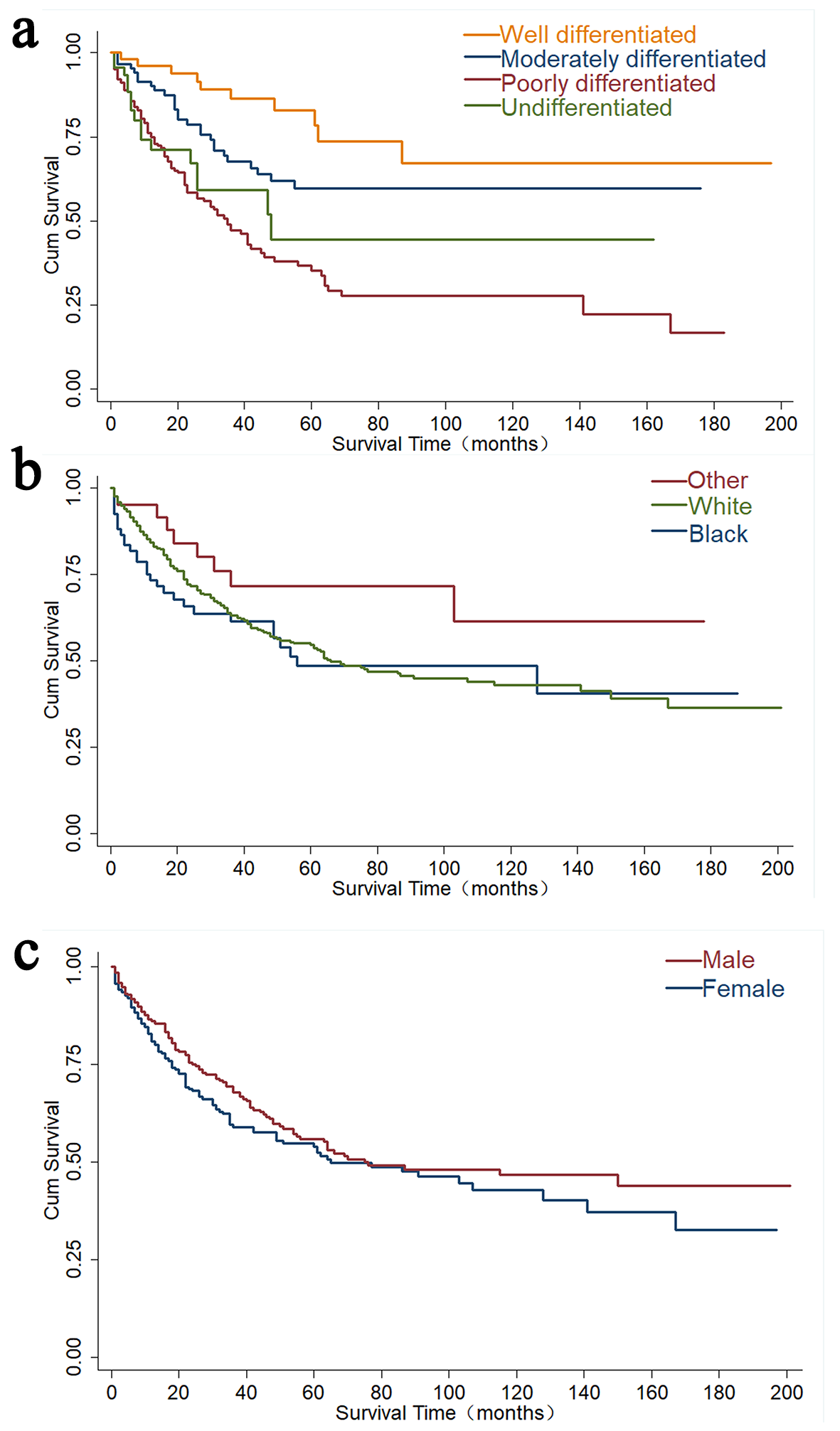

Supplement: Supplementary file 4 — Additional file 4: Figure S4. Long-Term Survival Outcomes using Kaplan–Meier’s analysis: (a) Long-Term Survival Outcomes in grade of gastrointestinal MANEC. Graph shows no difference in median survival between well and moderately differentiated disease (p = 0.069), difference in median survival between well and poorly differentiated disease (p < 0.001), difference in median survival between well and undifferentiated disease (p = 0.0017). (b) Long-Term Survival Outcomes in race of gastrointestinal MANEC. Graph shows no difference in median survival between white, black and other. (p = 0.136) (c) gender of gastrointestinal MANEC (no statistically significant difference). Graph shows no difference in median survival between male and female (p = 0.173). [file 12967_2020_2293_MOESM4_ESM.tif]
