# Supplementary material for: Gastrointestinal mixed adenoneuroendocrine carcinoma: a population level analysis of epidemiological trends
Source: J Transl Med. 2020 Mar 14;18:128. doi: 10.1186/s12967-020-02293-0 (PMC7071749; doi:10.1186/s12967-020-02293-0)
Supplement: Supplementary file 5 — Additional file 5: Table S1. Univariate cox’s proportional hazards model assessing factors associated with mortality after diagnosis of mixed adenoneuroendocrine carcinoma in appendix and cecum. [file 12967_2020_2293_MOESM5_ESM.docx]

**TableS1.** **Univariate Cox’s Proportional Hazards Model Assessing Factors Associated With MortalityAfter Diagnosis Of Mixed Adenoneuroendocrine Carcinoma In Appendix And Cecum**

|  |  | Appendix |  |  |  | Cecum |  |  |  |
| --- | --- | --- | --- | --- | --- | --- | --- | --- | --- |
| Risk Factor |  | HR* | Lower | Upper | P Value | HR* | Lower | Upper | P Value |
| Age at diagnose(years) |  |  |  |  |  |  |  |  |  |
| ≦60 |  | Referent |  |  |  | Referent |  |  |  |
| >60 |  | 1.52 | 1.04 | 2.20 | 0.02 | 1.0 | 0.49 | 2.01 | 0.99 |
| Race |  |  |  |  |  |  |  |  |  |
| Other |  | Referent |  |  |  | Referent |  |  |  |
| White |  | 1.88 | 0.46 | 7.60 | 0.38 | 1.16 | 0.27 | 4.93 | 0.84 |
| Black |  | 3.50 | 0.79 | 15.50 | 0.09 | 0.90 | 0.16 | 5.09 | 0.90 |
| Gender |  |  |  |  |  |  |  |  |  |
| Male |  | Referent |  |  |  | Referent |  |  |  |
| Female |  | 1.58 | 1.10 | 2.29 | 0.014 | 0.74 | 0.37 | 1.51 | 0.41 |
| SEER stage |  |  |  |  |  |  |  |  |  |
| Localized |  | Referent |  |  |  | Referent |  |  |  |
| Regional |  | 3.98 | 1.82 | 8.71 | <0.001 | 3.08 | 0.40 | 23.53 | 0.28 |
| Distant |  | 22.99 | 10.91 | 48.46 | <0.001 | 7.86 | 1.03 | 59.55 | 0.04 |
| Treatment |  |  |  |  |  |  |  |  |  |
| Surgery |  | Referent |  |  |  | Referent |  |  |  |
| No surgery |  | 5.71 | 2.46 | 13.25 | <0.001 | 2.26 | 0.76 | 6.77 | 0.14 |
| Grade |  |  |  |  |  |  |  |  |  |
| Poorly differentiated |  | Referent |  |  |  | Referent |  |  |  |
| Undifferentiated |  | 0.21 | 0.05 | 0.86 | 0.03 | 1.22 | 0.41 | 3.63 | 0.71 |
| Moderately differentiated |  | 0.18 | 0.07 | 0.44 | <0.001 | 0.93 | 0.34 | 2.49 | 0.88 |
| Well differentiated |  | 0.16 | 0.06 | 0.454 | <0.001 | & | & | & | & |
| Regional lymph nodes |  |  |  |  |  |  |  |  |  |
| Negative |  | Referent |  |  |  | Referent |  |  |  |
| Positive |  | 8.39 | 5.04 | 13.98 | <0.001 | 4.61 | 1.09 | 19.51 | 0.03 |
| Tumor size |  |  |  |  |  |  |  |  |  |
| ≦2cm |  | Referent |  |  |  | Referent |  |  |  |
| >2cm |  | 3.57 | 1.75 | 7.28 | <0.001 | 2.20 | 0.52 | 9.36 | 0.29 |

*HRs greater than 1.0 indicate a higher risk of death

& Since there is only one patient with a well differentiated disease, HR cannot be calculated
